# S65. INDEPENDENT COMPUTERISED COGNITIVE REMEDIATION FOR PSYCHOSIS: AN INVESTIGATION OF PATIENT EXPERIENCES

**DOI:** 10.1093/schbul/sby018.852

**Published:** 2018-04-01

**Authors:** April Hargreaves, Niamh Daly-Ryan, Rachael Dillon, Gary Donohoe

**Affiliations:** 1 Trinity College Dublin; 2 NUI Galway

## Abstract

**Background:**

Cognitive remediation (CR) is an effective therapy, shown to improve cognitive performance and functioning in patients with psychosis. We recently conducted a randomised control trial (RCT) demonstrating the effectiveness of a new computer based therapy, conducted with little therapist support. This study aims to assess the subjective experience of the participants in this trial.

**Methods:**

Twenty people with psychosis conducted a post-RCT questionnaire facilitated interview, which assessed their satisfaction with CR. Thematic analysis was then employed to identify common themes.

**Results:**

Three broad themes were identified, with participants reporting a predominantly positive experience of taking part in the therapy. In particular, participants reported improved cognition, improved positive self-regard, a development of life skills and a transfer of benefits to everyday life. Whilst there were reports of the therapy being difficult and tiring, patients expressed a positive attitude towards their therapist and a reluctance to see the therapy come to a close.

**Discussion:**

It is acceptable and beneficial for patients with psychosis to undertake independent CR therapy with reduced therapist contact.

